# The Formation of Collective Silk Balls in the Spider Mite *Tetranychus urticae* Koch

**DOI:** 10.1371/journal.pone.0018854

**Published:** 2011-04-14

**Authors:** Gwendoline Clotuche, Anne-Catherine Mailleux, Aina Astudillo Fernández, Jean-Louis Deneubourg, Claire Detrain, Thierry Hance

**Affiliations:** 1 Earth and Life Institute, Biodiversity Research Centre, Université catholique de Louvain, Louvain-la-Neuve, Belgium; 2 Unit of Social Ecology, Université Libre de Bruxelles, Bruxelles, Belgium; Vrije Universiteit, The Netherlands

## Abstract

*Tetranychus urticae* is a phytophagous mite that forms colonies of several thousand individuals. These mites construct a common web to protect the colony. When plants become overcrowded and food resources become scarce, individuals gather at the plant apex to form a ball composed of mites and their silk threads. This ball is a structure facilitating group dispersal by wind or animal transport. Until now, no quantitative study had been done on this collective form of migration. This is the first attempt to understand the mechanisms that underlie the emergence and growth of the ball. We studied this collective behaviour under laboratory conditions on standardized infested plants. Our results show that the collective displacement and the formation of balls result from a recruitment process: by depositing silk threads on their way up to the plant apex, mites favour and amplify the recruitment toward the balls. A critical threshold (quorum response) in the cumulative flow of mites must be reached to observe the emergence of a ball. At the beginning of the balls formation, mites form an aggregate. After 24 hours, the aggregated mites are trapped inside the silk balls by the complex network of silk threads and finally die, except for recently arrived individuals. The balls are mainly composed of immature stages. Our study reconstructs the key events that lead to the formation of silk balls. They suggest that the interplay between mites' density, plant morphology and plant density lead to different modes of dispersions (individual or collective) and under what conditions populations might adopt a collective strategy rather than one that is individually oriented. Moreover, our results lead to discuss two aspects of the cooperation and altruism: the importance of Allee effects during colonization of new plants and the importance of the size of a founding group.

## Introduction

In many species, individuals live in groups and form aggregates. It is recognised that individuals of those species may benefit from the presence of conspecifics, a concept broadly called the Allee effect [1]–[3]. Indeed, group living brings advantages both for the individual and the group as it provides easier access to food and mates as well as protection against predators [4]–[5]. It also presents disadvantages due to competition [4]. Aggregation at a chosen place for a long period is a prerequisite of social organization, because close interaction between individuals is only possible if they have frequent contacts [6].

Spatial distribution and spatio-temporal organization are important issues that determine the distribution of the species and the organization of groups. Patterns of movement are considered a key factor in the survival of most organisms [7]–[8]. Collective movements are ubiquitous in gregarious invertebrates and vertebrate species, including humans [9]. For animals foraging in groups, making decisions such as leaving the group to forage and/or to recruit other foragers to a food source often depends on social interactions among group members. Animals must move to find a new place to live (i.e. food shortage, finding a mate, climatic constraints). In some cases, they can benefit from collective dispersal because it implies that the new founding populations are large enough to benefit from potential Allee effects [1]–[3].

The consequences of dispersal on mites' demography and spatial distribution have been investigated in several studies on tetranychids. However, the behavioural mechanisms involved in collective dispersal in overcrowded conditions remain poorly understood. *Tetranychus urticae* (also called the two-spotted spider mite) has a very rapid population growth, short developmental time, a high birth rate and long adult survival [10]–[11]. This, together with the use of agricultural plants as a food source (more than 900 plants including field crops, horticultural crops, green house vegetables and ornamental plants [12]), has made this species a major pest. Consequently, research has focused mainly on the management and control of this species, whereas its behaviour, especially its social behaviour, has often been disregarded. Nevertheless, *T. urticae* is an ideal biological model for the study of individual non-eusocial behaviour and aggregate formation in overcrowded conditions. Indeed, this ubiquitous phytophagous mite, which lives in huge groups, quickly exhausts the host plant and must recurrently disperse to new host resources. The question is: What do individuals do when a group suffers from food shortage? *T. urticae* has different strategies to disperse: **1.** Dispersal by *active movement* –i.e by walking [13]–[15] **2.** *Phoresy* –i.e passive transport by another organism [16]–[18], **3.** *Aerial displacement* by air currents [19]–[21]. One important biological feature of *T. urticae* is its abundant silk production due to a continuous silk deposit while walking as reported by [22]. Silk threads are used as a physical support for locomotion [22]–[25] and can be used for aerial dispersal [20]. This individual passive displacement called ballooning is a mechanical kiting that many small species of spiders (Araneae), spider mites (Acari), and some moth larvae (Lepidoptera) use to disperse through the air [12], [20], [26]–[28]. Some of these ballooning spider mites (e.g. *Metatetranychus citri*, *Tetranychus pacificus*) were found to travel a few hundred meters [29]–[31] and might fly up to 3 km away [32], **4.** In *T. urticae*, a collective displacement seems to occur in conditions of overcrowding and food depletion on a host plant: The formation of *silk balls* that can be carried away by the wind or by a passing animal (anemochory or zoochory [33]). Different forms of silk balls have been described in *T. urticae*: balls can be an aggregate of mites embedded in silk on the plant [34] or they can hang at the end of a silk thread [35]. As the population peaks, the mites congregate on leaf tips, spin silk threads, and form small masses from which mites can be observed to be carried aloft in light breezes [36]. The aerial dispersal of these aggregates may be an important element in the spatial dynamics of *T. urticae* populations [36]: it might explain the sudden outbreak of large spider mite populations in crops that, apparently, were previously uninfected. Until now, the processes involved in the formation of mites' aggregate and silk balls are still unknown.

Despite the role of silk threads, aggregate formation and dispersal for a colony of mites have been poorly studied. Studies dealing with silk on spiders and caterpillars have shown that silk threads are a means for communication among individuals [37]–[39] and are used for collective dispersal. In spider mites, silk threads might trigger individuals' displacements; they could play a role in the control of recruitment process and collective movement [25], [40]. The silk-producing (spinning) habit and other peculiarities of the Tetranychidae, i.e. male-haploid genetic system, gregariousness and life history, may be related to the evolution of their sociality [41].

We present here, for the first time, a study of the emergence and growth of collective silk balls. Silk threads could be the key element for communication among individuals, recruitment process and the ball formation. Our study aimed: (i) to understand the mechanisms that lead to the formation of the silk balls, (ii) to study the dynamics of balls' formation, and finally (iii) to characterize the balls' content. Understanding how these collective balls emerge and change is an important question as dispersal is a transient crucial phase in a colony life.

## Materials and Methods

### Mite strain

The strain (LS-VL) of *T. urticae* was collected in October 2000 from roses in Ghent, Belgium [42]. In the laboratory, two-spotted mites were reared on bean plants (*Phaseolus vulgaris* L.), a preferred host plant [43]. Stock breeding was maintained in a climate room under 26°C, with a relative humidity of 50–70% and a photoperiod of L16:D8.

### Plants infestations

Bean plants used in this experiment were reared under standardized conditions in a growth chamber (20.6°C, 24% R.H.). Plants with a height between 10.5 and 11 cm, with two young leaves were used. During the whole experiment, only the two first leaves (cotyledons) were kept on the plant, while all the other shoots were cut daily.

**Figure 1:**
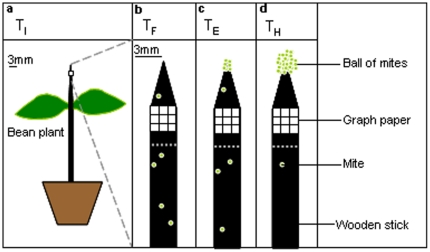
Experimental set-up. a). Plant infestation (15 mites on each leaf) **T_I_**. b) Ascending flows of mites on the stick **T_F_** c). Emergence of the ball **T_E_** and d). Harvesting of the ball **T_H_**.

A black wooden stick (25 cm high, 3 mm in diameter) was attached to the stem of plants (Fig. 1). The stick was buried in the soil, 20 cm high and attached to the stem with a piece of plastic wire. A square piece of graph paper (2×2 mm) was fixed on the stick to scale the surface of the ball. The presence of this paper did not prevent mites from climbing up on the wooden stick. Mites were collected using a brush with one hair. Thirty adult females randomly chosen in the rearing stock were introduced on the test plant (15 on each leaf) (infestation time, T_I_) (Fig. 1a). Then, bean plants were placed individually in a cage (50×50×40 cm) under standardized conditions (26°C, 33% RH). The population of mites was allowed to grow on the bean plant. Once the first mites arrived on the stick, the number of mites climbing to the top of the stick (flow of ascending mites, T_F_, N = 18, Fig. 1b) was counted over a period of 10 minutes, twice per day (at 8h15 and 14h) until the removal of the ball at about day 18 (harvesting time, T_H_, N = 15).

### Growth dynamics

The apex of the wooden stick was observed every hour to detect the ball's formation (emergence time, T_E_) (Fig. 1c). We considered that a ball was formed when there were at least ten mites permanently aggregated on top of the wooden stick. From there onwards, photos were taken at regular time intervals (every hour from 8 to 17 h). The camera used was a Canon EOS 450D equipped with a canon macro lens (EF, 100 mm, 1∶2.8, USM). The scale of the graph paper allowed us to compare volumes (if considering balls as spheres) for the same ball. With the software Image J ®, the perimeter and the surface of the balls was calculated over time. The day of ball formation (T_E_) was noted and the ball was “harvested” the day after its formation at 14 h (T_H_) (Fig. 1d). To collect the ball, the wooden stick was carefully removed from the plant and thereafter the ball was collected from the stick end with tweezers.

### Content of balls by layer

Once removed from the wooden stick, the ball was placed on a glass lens (32 mm in diameter) for about 30 minutes. Mites situated on the surface of the ball left the ball and walked on the lens. Once every mobile mite of the ball was on the lens, the “core” of the ball (trapped or dead mites) was transferred onto a new glass lens. The number of mites was then counted for the two glass lenses. In this way, mites found on the first glass lens were those from the outer layer of the ball and mites counted on the second one were from the inside layer. The number of mites, the stage (immature or mature) and the state (dead or alive) were noted.

### Data analysis

Data obtained during our observations were not always normally distributed. Therefore, both parametric and non-parametric tests were used in our analysis. A linear regression was used to determine the relationship between two variables (Y and X), and the non-linear least squares method was used when the relationship was not linear. Curve fitting adjustments and the logistic regression were performed with Matlab. A paired t-test was used to determine difference between survival rates of mites inside balls. The Kruskal–Wallis (KW) one-way analysis of variance by ranks was used to test the equality of medians among groups. The Kolmogorov-Smirnov test (KS) was used to determine if two datasets were significantly different. The Mann-Whitney Test was used to determine whether the means of immature and mature (in balls and between layers) were equal.

Tests were performed using GraphPad Prism version 5.01 forWindows, GraphPad Software, San Diego, California, USA (http://www.graphpad.com).

## Results

### Ascending Flow

Although the ascending flow (number of mites climbing upwards during 10 minutes) was measured twice per day, we assume that it is representative of the general dynamics of ascension. The flow was measured 1.5 centimetres below the top of the stick, and every mite observed to climb up the stick reached the top. Therefore, we used the cumulated flow (x) as an estimate of the number of mites that have reached the top since the beginning of the experiment. Evidently, the actual number of mites that had reached the top was much larger, but we assumed that it was proportional to the measured cumulated flow. The actual flow was the speed at which the cumulated flow increases (dx/dt). For each experiment, the flow at a given time (dx/dt) was proportional to the cumulated flow (x), i.e. the number of mites having reached the top since the beginning of the experiment (linear regressions with equation (1) showed an R^2^ of 0.75 (0.57; 0.91) (median and first and third quartiles).
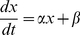
(1)


Parameters *α* and *β* were respectively the recruitment factor (*time* ^-1^), and the spontaneous flow of mites before the recruitment had started (*nb of mites* * *time* ^-1^). The fact that the flow of passing mites increased linearly (*α*x) with the number of mites that had already passed strongly suggested the existence of positive feedback. This positive feedback typically resulted from a recruitment process. Each passing mite increased the probability of climbing up for the subsequent mites, which led to an amplification of the flow. Therefore, the number of mites that had reached the top (the cumulated flow) should have increased with time in a non-linear way. The relationship between the cumulated flow and time (equation 2) was obtained by integrating equation 1. We fitted this equation to the data of each experiment and found a tight correspondence (non-linear least-squares method, R^2^ always superior to 0.97), which confirmed the existence of a recruitment process.
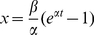
(2)


**Figure 2:**
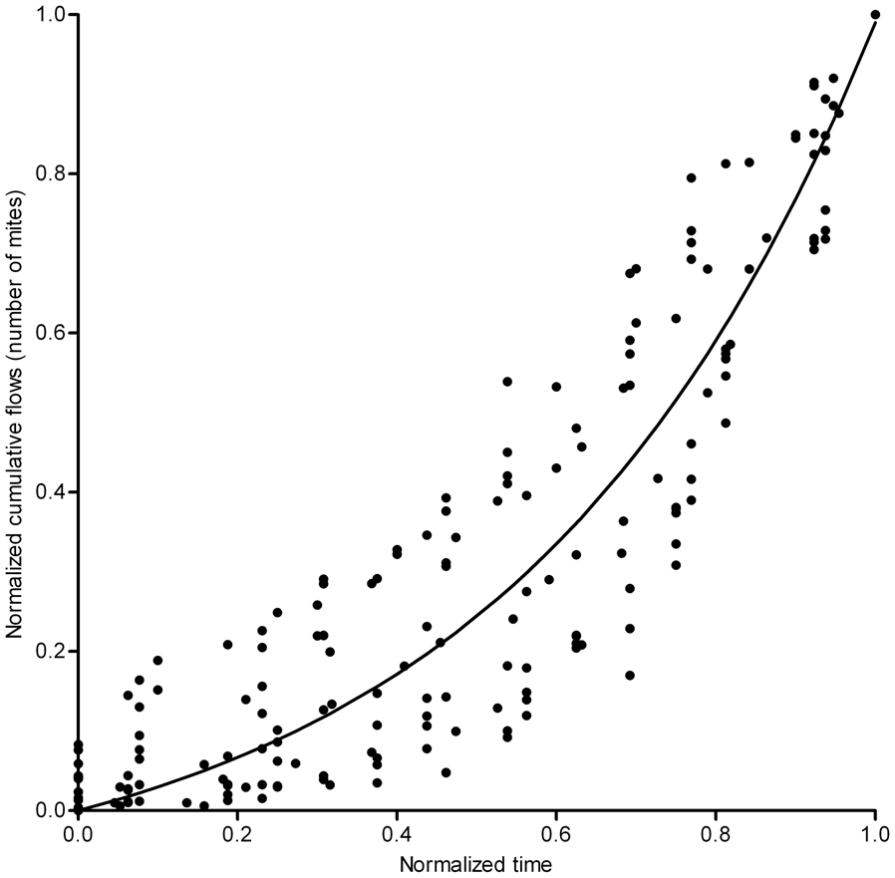
Increase of the cumulated flow according to time. The data of each experiment was normalised according to maximum time and maximum cumulated flow. The fitted curve (Equation 2) has parameters *α* = 2.231(1.959; 2.502) and *β = *0.266 (0.223; 0.308).

The values of *α* and *β* varied from experiment to experiment, reflecting simply that in some experiments, the balls emerged faster than in others. They ranged between 0.0018 and 0.0452 for *α*, and between 0.1927 and 5.0558 for *β*. The flow was measured until the silk balls were removed (one day after their emergence at 14 h); therefore the time of observation varied from experiment to experiment. The global dynamics were obtained by normalising the time according to the maximal time, and the cumulated flow according to the maximal cumulated flow. The global dynamics followed the same pattern as each experiment separately (Eq. 2, non-linear least squares method, R^2^ = 0.90, Fig. 2). This meant that the slower the increase of the cumulated flow, the longer the experiment took. This suggested a possible link between the time of formation of the ball and the cumulated flow at that time.

### The emergence of silk balls

**Figure 3:**
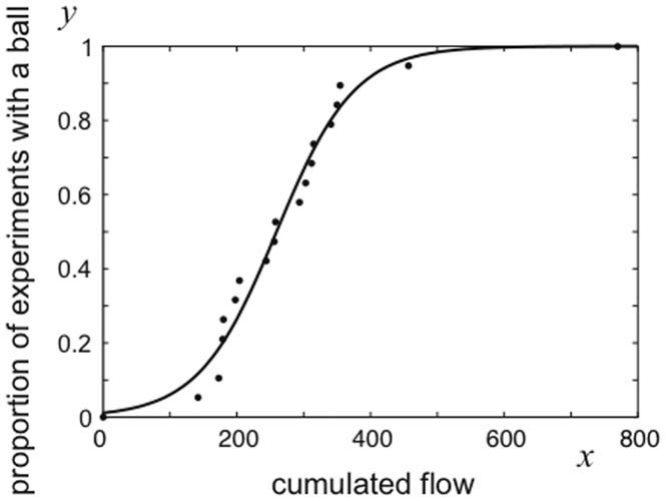
Proportion of experiments in which the balls emerged (*y*) after a cumulative flow of *x* mites. The curve presents a threshold (*σ* = 259.4 mites). A logistic function (solid line) was fitted to our experimental data (dots) with a logistic regression.



For the first few days, the mites that were recorded to climb up the wooden stick (ascending flow) would reach the top and immediately begin to climb down. When the silk balls emerged, however, some of the mites started to get trapped at the top of the stick, favouring the growth of the silk ball. The relationship between the moment of emergence of the silk balls and the cumulated flow appeared when looking at the proportion of the experiments in which the ball has emerged, as a function of the cumulated flow (Fig. 3). The cumulated flow was proportional to the number of mites that had passed to the top of the stick since the beginning of the experiment. Since mites always spin silk while walking, the cumulated flow was also an indicator of the quantity of silk that had accumulated on the top of the wooden stick. The curve was clearly sigmoidal, meaning that the balls emerged on top of the wooden stick when a threshold number of mites had passed (and not necessarily stayed) at the top of the wooden stick. This suggested that there needed to be a critical quantity of silk on the top for the first mites to stay and start forming the ball. In order to quantify the phenomenon, we performed a logistic regression (equation 3) with the cumulated flow as the independent variable and the presence or absence of a ball as the binary dependent variable. The logistic curve (equation 3, Fig. 3) expressed the proportion of experiments in which a silk ball was present (y), according to the cumulated flow (*x*).

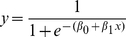
(3)The coefficients *β_0_* and *β_1_* were estimated with the logit regression (*β_0_ +* s.e. = -4.4553+0.5062; *β_1_* + s.e. = 0.017+0.0019). The threshold (*σ*) of this function was calculated as the cumulated flow for which half of the experiments had a ball, that is *σ = - β_0_*/*β_1_* = 259.4. We calculated the probability of emergence of a ball as a function of the cumulated flow (equation 4, where *z* is the survival curve, *z = *1-*y*). This probability increased with time and approached an asymptotical value of *β_1_ = *0.017. Therefore, a prediction resulting from this model was that the ball eventually emerged on the apex of a plant, provided that mites continue to climb upwards. 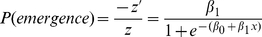
(4)


### Growth dynamics

**Figure 4:**
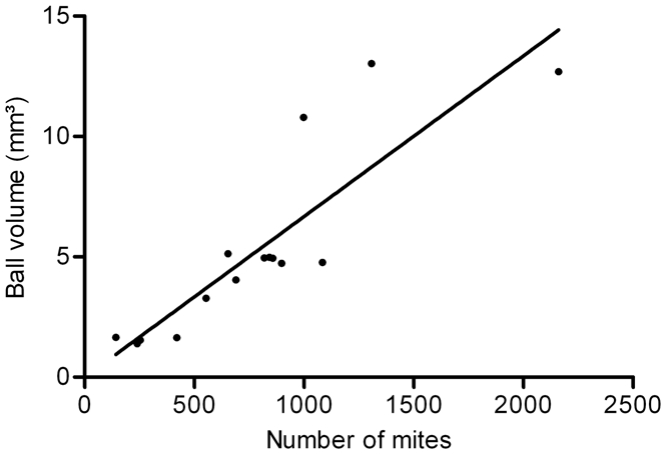
Correlation between final volume of balls (if considering balls as spheres, volume on T_H_-14 h) and number of mites (after dissection). The volume was correlated to the number mites (p<0.005, N = 15). The equation was Y = 0.0067x with R^2^ = 0.76 (N = 15).

**Figure 5:**
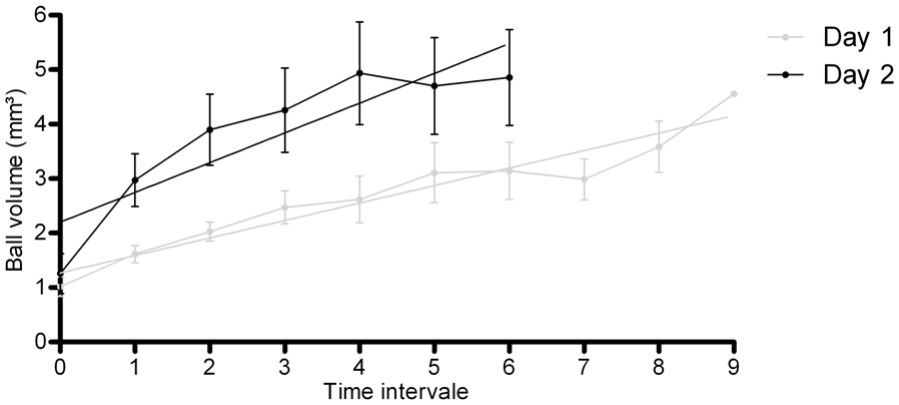
Growth of balls volume for the two consecutive days (two phases). Both phases were roughly linear (phase 1: y = 0.321x+1.269, R^2^ = 0.924 and phase 2: y = 0.546x+2.201, R^2^ = 0.79). The slope of phase 2 was steeper than that of phase 1 (F-tests comparing slopes: F_1,13_ = 4.72, p = 0.049) due to a higher flow and possibly because fewer mites came down.

As the cumulated flow (number of mites reaching the top) increased non-linearly, we expected the ball also to grow non-linearly. The final volume of balls (T_H_, 14 h) was significantly linearly correlated to the number of mites (after dissection). The equation was T_H_ = 0.0067x with R^2^ = 0.76 (N = 15) (Fig. 4). This correlation allowed us to use estimations of the volume of the balls as an indicator of the number of individuals in the ball. When looking at the growth of the ball through time, two phases of growth were observed. The two growth phases (corresponding to the two consecutive days of observation) were separated by a disintegration phase during the night. Both phases presented linear growth (phase 1: y = 0.321x+1.269, R^2^ = 0.924 and phase 2: y = 0.546x+2.201, R^2^ = 0.79, Fig. 5). However, the slope of phase 2 was steeper than that of phase 1 (F-tests comparing slopes: F_1,13_ = 4.72, p = 0.049), meaning that in phase 2, the ball increased faster. Two possible explanations (non-exclusive) can be raised. Either there were more mites climbing up during phase 2, or there were fewer mites climbing down. We only had quantitative observations concerning the ascending flow, so we can only test the first of the two explanations. The slope of each experiment (for each phase) was highly correlated to the flow at that moment (Spearman correlation coefficient r = 0.55, p = 0.0005). Therefore, we can conclude that the balls grew faster in the second phase, due to a higher ascending flow.

### Content of balls by layer

To characterise the content of the balls, in terms of the number of mites, the stage (mature or immature), the state (dead or alive), and the layers (outer and inner parts), was essential to better understand how individuals formed the ball over time. Because the inner layer was formed by individuals before the outer one, the stage or state of individuals could be linked to the layers (existence of individuals initiating the ball formation). The risk of dying during the formation of the ball should also vary according to the layers or the ball volume.

A total of 15 balls were dissected. The number of mites contained in each ball showed a normal distribution (from 141 to 2162 ind, mean: 793.5 ind ± 503.1, Kolmogorov-Smirnov test, p>0.10, KS = 0.151, N = 15). The outer part of each ball (outer ball, 56.9%±49.5 of the entire ball) was composed of mites that have not been trapped in silk threads, whereas the inner part (inner ball, 43.1%±16.7 of the entire ball) was formed by mites trapped by silk.

Most mites inside the entire ball were immature individuals (mean: 98.9%±1.1, N = 15). The number of immature mites was higher than mature ones regardless of the layer (for the outer and inner parts, Mann Whitney test, U = 0.00, p<0.0001, N = 15). The medians of the number of immature and mature were not statistically different between layers (for mature individuals, Mann-Whitney test, U = 108, p = 0.867, N = 15 and for immature individuals, Mann-Whitney test, U = 81.5, p = 0.206, N = 15).

Entire balls were mainly composed of alive mites (67.8%±15.9, 67.8%±15.9, χ^2^ = 161.4, df = 14, p<0.0001, chi-square test comparing percentages of alive and dead mites in balls, N = 15). The core of the balls was essentially composed of dead mites, as is shown by the percentage of dead mites in each part of the ball (2% of dead mites ± 2.1 for the outer ball and 68% of dead mites ± 20.8 for the inner ball) and was significantly different between the two parts (t-test comparing the proportion of dead mites per layers, t = 12.01, df = 14, p<0.0001, N = 15). The proportion of survivors was not correlated with the size of the ball (R^2^ = 0.024, N = 15) and not correlated to the stage of individuals (t-test comparing the survival rate between immature and mature stages, t = 1.224, df = 26, p<0.05, N = 14). Table 1 shows the average number of individuals (calculated from N = 15 balls) contained in each of the two layers, according to their stage (mature or immature) and their state (dead or alive).

**Table 1:** Number of individuals (mean ± sd, N = 15) contained in a ball.

Individuals	Outer ball	Inner ball
Mature	6±9	4±4
Immature	418±242	366±187
Alive	417±247	109±113
Dead	7±7	261±281
Total per layers	424±249	370±371
Total in the ball	794±502	

The stage (mature or immature) and the state (dead or alive) were noticed per layers (outer or inner part).

## Discussion

The high reproductive rate of spider mites allows the colony to quickly multiply and over exploit their habitat. Therefore, they have to face both food shortages and host plant desiccation [44]–[45]. This forces them to migrate and colonise new plants [35], [46]–[47]. In our study, we observe mites during their migration and we cast light on the mechanisms involved in the formation of silk balls. This collective form of migration is mentioned in many papers, but always as qualitative observations. This is the first attempt to understand the mechanisms that underlie ball formation. Our results provide enough insight to reconstruct the key events that lead to the formation of silk balls.

The necessary condition for the whole process to be triggered seems to be food shortage and a high density of mites. Once the plant is exhausted, the mites start climbing toward the apex of the plant. This ascension is self-amplified: we show that the mites' tendency to climb up the wooden stick increased with the number of mites having ascended in the past. This correlation is typical of a recruitment process most probably due to the accumulation of silk threads. Indeed, [25] showed that migrating females can simply follow trails (silk threads) left by preceding individuals. The positive phototaxism of *T. urticae* [48] leads mites towards the apex of a stick mimicking the plant stem. During migration to the apex, mites would contribute to the growth of webbing through the addition of silk threads on the wooden stick. Before the formation of the ball, the mites that reached the apex immediately started their descent back to the plant: they did not stay at the apex. The amount of silk deposited on the stick was therefore doubled. The increase of the silk quantity would stimulate the departure of new mites, which would in turn deposit silk. This positive feedback is likely to be underlying the recruitment process that appears in our experiments. In social insects such as ants, recruitment processes are well known: one forager discovers an important food source, recruits nest-mates, which in turn recruit still more foragers [49]. This can be due to chemical signals (trail pheromones) that stimulate the departure of recruits as shown in ants, termites and some bees [49]–[50]. Silk threads are also used for recruitment as shown in spiders and social caterpillars [51]-[53]. Recruitment allows groups to exploit en masse random discoveries [50], [54]–[57], or to form clusters [58]–[59]. In *T. urticae*, this recruitment promotes the aggregation of mites into one unique ball, and seems to prevent the random dispersal of individuals. Further laboratory studies on silk ball formation need to be done using more than one single stick. Indeed, in natural conditions, there would be many stems upon which the mites could climb. It will be interesting to observe whether the dynamics of ball formation as well as the number of emerging balls will differ when the mites have to choose between different wooden sticks.

The ascent and descent of the mites continues for a few days until at some point, the mites that reach the apex start spending more time there, which leads to an accumulation of mites and silk. This is when the ball emerges. This emergence seems to take place when a threshold number of mites have passed at the apex since the beginning of the whole process, following a quorum-type [60] response function. Spider-mites systematically spin silk when they move [22]. Therefore, the amount of silk that accumulates at the apex is directly proportional to the total number of mites that have reached it. It is known that silk has a retentive effect on *T. urticae* [25]. Our hypothesis is therefore that at some point, the quantity of silk at the apex is sufficient to retain the mites that reach the top: this is when the ball emerges. Our results predict that a silk ball will eventually emerge, as long as mites keep climbing up the stem. In their natural habitat, mites can stop climbing up a stem for many different reasons (there are not enough mites, there are too many stems, the mites leave the plant by another means…). This explains partly why silk ball formation is only observed under certain conditions that include high densities. Quorum responses are a ubiquitous feature of consensus decision-making and previous work proved their importance in generating aggregation, cohesion and decision accuracy [60]–[61].

Once formed, the silk ball seems to have its own life dynamics characterized by alternating phases of daily growth and nocturnal decrease: first, the ball grows due to the ascending movement and aggregative behaviour of the mites; this increase rate being higher in the second day because of silk threads accumulation on the wooden stick. This is likely to be related to *T. urticae* circadian rhythm during which mite populations move up in the early afternoon and migrate to the bottom of plants during the night [48]. This rhythm seems to be partly endogenous, since mites continued to move up plant stems for several days after being moved from fluctuating conditions [48]. Therefore, during the formation of the ball, two processes reinforce or counteract each other alternately: the amplification process resulting from the addition of silk threads drives individuals to form balls and the circadian response to light (alternation of negative and positive phototropism) that reinforces the cohesion of balls during the day and reduces it during the night. The behaviour of the mites during migration might result from the balance between these two trends.

Once formed, the balls are not firmly attached to the apex of the plant. In the field, the wind or a passing animal would be sufficient for the dispersal of the ball. In passive airborne dispersal such as individual ballooning or collective silk balls, the distance and the direction of travel are largely determined by the air currents [20]. Aerial displacements may be tightly linked to fitness because of direct mortality risks related to the potential (uncontrollable) dispersal distance [62]–[63] and the spatial availability of other host-plants. Individual ballooning or collective balls in tetranychid mites is the result of an active behaviour that enhances the probability of mites being carried aloft from plant surface [46], [64]. It could be possible that with the combined protection of aggregation and silk threads, ball-dispersed eggs could hatch into an optimal environment for the young larvae. The study of the balls “life-time” seems essential to complete the understanding of this collective behaviour. Experiments are ongoing in our laboratory to measure the survival chances of a group of mites transported in a ball. A ball seemingly dead (an aggregate of desiccated mites) could infect a new plant through the survival of some eggs or individuals inside the aggregate. It would be also interesting to study how far and under which conditions (e.g. wind speed) such balls can disperse.

The dissection of the balls showed that they were composed mainly of young stages. Many immature mites also remained on the bean leaf (personal observation). However, a quantification of the demographic composition of mites on the leaf is missing to assess whether mites are joining the ball at random or as a function of their stage. The new founding populations on a new host would therefore allow exponential growth. Indeed, starting with such a non-stable age structure population enhances population growth well over the prediction of the classical exponential model [11].

The dissection of the balls also showed that more dead mites are found in the inner part of the balls. Indeed, the first mites initiating the ball are trapped within a dense silk network and can die of hunger and suffocation. Some of them are females that lay eggs in the ball. Only recently arrived individuals are still alive. Therefore, each ball is composed of one inner part composed of a higher number of dead mites (and some eggs) than in the outer layer. This feature could simply be a side effect due to laboratory conditions in which there is no wind or passing animals, and the ball is allowed to stay indefinitely on the wooden stick. It could also be a natural phenomenon, raising interesting questions about its functionality. Studies are ongoing in the laboratory to better understand how individuals die and what benefits are provided to the survivors. The production of a collective structure such as the ball could be influenced by the degree of relatedness between individuals. Ongoing genetic studies using fourteen microsatellite loci, aim at estimating the degree of relatedness of individuals inside the ball. Because dead individuals were found in the ball core, kin selection is thought to interfere with the process. The genetic characterization of both individuals being part of the ball and those remaining on the plant leaf will help to understand the dispersion strategy of *T. urticae*.

Surely, the formation of balls represents an energy cost (mobility and silk deposit) for mites, but it is likely that the benefits outweigh the costs. Our hypothesis is that mites dispersing by silk balls could benefit from Allee effects. Silk balls can be considered a form of aggregation with protective and dispersal roles. Among the possible benefits of founding populations in a group, a decreased predation threat and increased foraging efficiency are the most commonly accepted Allee effects [65]–[66]. Aggregation may also affect the physiology of individuals by influencing the microclimate. For instance, it contributes to water conservation by reducing the surface area: increasing volume ratio of the group and limiting water loss [67]–[68] as shown in the hissing-cockroach mite *Gromphadorholaelaps schaeferi* [69], in the dust mite *Dermatophagoides pteronyssinus* [70] and in some eusocial Hymenoptera [71]–[72]. Clustering is a common reaction in acarines as they undergo dehydration as a means to counter water stress [70]. [34] claimed that mites were able to vacate silk cocoons formed on plants from which water had been withheld.

Insect societies provide us with remarkable examples of sophisticated collective activity including nest construction, complex foraging strategies, and collective decision making [49], [57], [73]–[76]. One of the most visually striking examples is the physical structures composed of individuals that have linked themselves to one another. These “self-assembled” structures [77]–[78] are a product of the process of self-assembly (i.e. army ant bivouac). The silk balls that are the subject of this study are a particular case of a wide array of self assembled structures found in *T. urticae*. For example, under forced migrations from a heavily damaged, isolated food substrate (bean leaves on soaked cotton), [79] observed that the migratory activity of *T. urticae* took the form of aggregations of visibly excited mites. In such groups, the mites produced web relatively intensively and formed a bridge between bean leaves made from mites and silk threads. Other structures are hanging “ropes” composed of silk and individuals entangled in it ([15], personal observation). All of these self assembled structures seem to share silk as the key element both for ensuring the cohesion between individuals and for promoting the arrival and withholding of individuals due to their attraction and retention to silk.

Under certain environmental and group conditions, in addition to the fact that mites follow trails left by preceding individuals, *T. urticae* form a silk ball. The ball is a way of collective dispersal emerging from interactions among individuals. This study is focused on the positive feedbacks that result from these interactions (i.e. recruitment, existence of thresholds). However, silk ball formation is regulated by an interplay between these positive feedbacks and a series of negative feedbacks such as overcrowding at the top of the stem, saturation of the flow on the stem, or limited size of the population. A quantification of all of these elements would be useful to carry out in the future: this would allow the building of a dynamical model that would provide not only a thorough quantitative description, but also a better understanding of the rules that govern the collective migration dynamics of *T. urticae*.

Advantages and disadvantages of this mode of dispersal must be studied in the future in order to further understand the role of collective silk balls in the colonization of new habitats.

Elucidation of these collective behaviours will allow a better understanding of the social bonds within a colony and their potential influence on aggregation, dispersal, and web building in a mite colony. Understanding how these collective balls emerge and evolve is an important question and should contribute to a better understanding of group behaviour and subsocial organization in this species.
